# Seroprevalence and Risk Factors for *Toxoplasma gondii* Infection in Women of Reproductive Age in Nigeria in 2018

**DOI:** 10.4269/ajtmh.24-0107

**Published:** 2024-09-10

**Authors:** Dawn Blackburn, Nwando Mba, William Nwachukwu, Hong Zhou, Andrew Hill, Andrew Abbott, Nishanth Parameswaran, Samuel Awala, Stacie Greby, Matthias Alagi, Nnaemeka C. Iriemenam, McPaul I. Okoye, Mahesh Swaminathan, Jeffrey W. Priest, Diana Martin, Anne Straily, Chikwe Ihekweazu

**Affiliations:** ^1^ Division of Parasitic Diseases and Malaria, U.S. Centers for Disease Control and Prevention, Atlanta, Georgia;; ^2^ Epidemic Intelligence Service, U.S. Centers for Disease Control and Prevention, Atlanta, Georgia;; ^3^ Nigeria Centre for Disease Control and Prevention, Abuja, Nigeria;; ^4^ Institute of Human Virology, Abuja, Nigeria;; ^5^ Division of Global HIV and TB, U.S. Centers for Disease Control and Prevention, Abuja, Nigeria;; ^6^ Division of Foodborne, Waterborne, and Environmental Diseases, U.S. Centers for Disease Control and Prevention, Atlanta, Georgia

## Abstract

Congenital transmission of *Toxoplasma gondii* can occur when a woman becomes infected for the first time during or just before pregnancy. *Toxoplasma gondii* in the fetus can lead to miscarriage, stillbirth, ocular or neurological abnormalities at birth, or progressive visual, hearing, motor, and cognitive deficiencies. The national seroprevalence of *T. gondii* infection in Nigeria was previously unknown. The 2018 Nigeria HIV/AIDS Indicator and Impact Survey collected demographic, socioeconomic, and HIV-related data and stored blood specimens with consent for future analysis for other pathogens of public health importance. We evaluated toxoplasmosis seropositivity and risk factors in a sample of 44,269 women of reproductive age (WRA) between 15 and 44 years. The national *T. gondii* seroprevalence among WRA was 26.8% (95% CI: 25.8–27.7%). We found that WRA from all 36 states and the Federal Capital Territory had *T. gondii* exposure. Seroprevalence was higher in 25- to 44-year-olds than in 15- to 24-year-olds. A similar proportion of pregnant and nonpregnant women were seropositive. Increased odds of seropositivity were associated with unimproved toilet facilities and drinking water sources, being in a higher wealth quintile, and primary and secondary education compared with no education. Decreased odds of seropositivity were associated with living in an urban area and owning livestock. This study provides the first-ever national seroprevalence estimate for WRA in Nigeria. Although information on known risk factors for toxoplasmosis (e.g., consumption of undercooked meat, cat ownership) was not collected, future studies could further investigate potential risk factors to inform the development of effective toxoplasmosis prevention measures.

## INTRODUCTION

Toxoplasmosis is caused by an obligate single-celled intracellular protozoan parasite, *Toxoplasma gondii*, that can infect many species of warm-blooded animals, including humans. Most infected immunocompetent individuals are asymptomatic or experience a self-limiting, nonspecific illness. However, ocular infection can sometimes lead to visual impairment. Differences in prevalence and severity of disease in individuals are driven, in part, by the genotype of the parasite.^1^ Immunocompromised individuals may develop severe disease that can be fatal.^2^

The only known definitive hosts for *T. gondii* are felids, which shed oocysts in their feces.^2^ Intermediate hosts such as birds, rodents, pigs, or sheep become infected after ingesting oocysts in the environment that may be in contaminated soil, water, or plant material. After ingestion, oocysts transform into tachyzoites, which localize in muscle and neural tissue and develop into tissue cysts (bradyzoites).^3^^,^^4^ Infection in humans can occur via ingesting these tissue cysts in undercooked, contaminated meat such as pork or lamb; via ingesting *T. gondii* oocysts from the environment, such as through contaminated food or water or via unwashed hands, such as after cleaning a cat’s litter box when the cat has shed *T. gondii* in its feces or after gardening where soil has been contaminated by cat feces containing the oocysts. Rarely, transmission through solid organ transplantation or blood transfusion may occur.^3^^–^^5^

Congenital transmission can occur when a woman becomes infected for the first time during or just before pregnancy, although reactivation in immunosuppressed women can also result in transmission to the fetus.^3^ Congenital transmission can result in miscarriage or stillbirth. A small percentage of infected newborns have serious ocular or central nervous system abnormalities at birth. Many infants are born with subclinical infection but will subsequently develop signs or symptoms of congenital toxoplasmosis, which may cause progressive visual, hearing, motor, and cognitive deficiencies.^3^^,^^4^ The global incidence rate of congenital toxoplasmosis is estimated to be 1.5 cases per 1,000 live births and the global burden of congenital toxoplasmosis has been estimated to be 1.20 million disability-adjusted life years per year.^6^

Women who are infected with *T. gondii* before pregnancy (i.e., IgG seropositive) have little risk of transmitting the infection to their fetus during subsequent pregnancies, even when rechallenged.^3^ Understanding the seroprevalence among women of reproductive age is one component that can be used to estimate the risk of congenital transmission in a population.^6^ A prior systematic review examining studies across 17 Nigerian states estimated the overall pooled prevalence of *T. gondii* infection as 32.92%, with a significantly higher pooled prevalence of 40.25% among pregnant women.^7^ However, the national seroprevalence of *T. gondii* infection in Nigeria among women of reproductive age (WRA) is unknown.

National individual disease-specific serosurveys are often not feasible. Multi-disease serologic surveillance maximizes resources by integrating testing for several diseases within a single survey.^8^ The 2018 Nigeria HIV/AIDS Indicator and Impact Survey (NAIIS) was a cross-sectional population-based household survey designed to estimate HIV burden at the national and state levels. The Nigeria Multi-Disease Serologic Surveillance using Stored Specimens project tested stored samples from NAIIS for other pathogens of public health importance (e.g., toxoplasmosis). The objective of our study was to determine the estimated seroprevalence of *T. gondii* in WRA (defined as women aged 15–44 years) in Nigeria and identify associated risk factors for infection, using NAIIS specimens and data. This would allow for an improved understanding of toxoplasmosis epidemiology, which may provide information for the evaluation of disease control and prevention programs and public health action, reducing the risk of *T. gondii* infection and congenital transmission among WRA.

## MATERIALS AND METHODS

### Survey design and sample collection.

The nationally representative 2018 NAIIS was led by the government of Nigeria and was conducted between July and December 2018, including collection of blood specimens, survey data and informed consent. The NAIIS used a two-stage stratified cluster sample design, selecting enumeration areas (EAs) followed by households. The first stage EA sampling units were stratified by 36 states and the Federal Capital Territory (FCT) and were selected systematically with probabilities proportionate to size based on the 2006 census. For the second stage, households were chosen within each selected EA through systematic sampling. All eligible adults (aged 15–64 years) were sampled within each selected household. Detailed methods have been described elsewhere.^9^

Women of reproductive age were sampled within the six geopolitical zones of Nigeria and two age groups (15–24 and 25–44 years) for a total of 12 strata. The ranges for the two age groups were determined based upon the risk of pregnancy-related vaccine preventable diseases (Congenital Rubella Syndrome and maternal and neonatal tetanus). There were 76,100 WRA with NAIIS stored dried blood spot (DBS) specimens available from participants who consented to future testing. Testing of these samples was divided into three tranches. For tranche 1, a minimal sample size was selected to address priority questions for tetanus and rubella. Tranche 2 included WRA from the 11 states that participate in the U.S. President Malaria’s Initiative (Cross River, Zamfara, Nasarawa, Sokoto, Bauchi, Benue, Ebonyi, Oyo, Kogi, Akwa Ibom, Kebbi). Tranche 3 included WRA as part of further testing of a selection of remaining stored specimens, including 1,720 HIV-positive adults and a selection of HIV-negative adults who were frequency matched with HIV-positive adults at a ratio of 1:4 based on 5-year age groups, state, and sex.

### Laboratory testing.

To obtain *T. gondii* serological data in Nigeria, we used a multiplex bead assay (MBA) to measure antibodies against the SAG2A antigen in DBS specimens. SAG2A antigen has been previously targeted in serological studies for *T. gondii* infection.^10^^–^^13^ The sensitivity and specificity of testing for antibodies against the SAG2A antigen by MBA has been estimated to be 100%.^14^

Punches measuring 6 mm were taken from Whatman 903 Protein saver DBS cards (Sigma-Aldrich, St. Louis, MO), protein was eluted, and the sample was diluted to a final serum concentration of 1:400 in Buffer B (phosphate-buffered saline pH 7.2 plus 0.5% casein, 0.8% polyvinylpyrrolidone, 0.5% polyvinyl alcohol, 0.3% Tween 20, 0.02% sodium azide, and 3 *µ*g/mL of *Escherichia coli* extract).^15^ Samples were assayed in single wells for total IgG antibodies as described previously.^16^

The SAG2A antigen bead coupling conditions have also been described previously.^17^ Data from the MAGPIX were reported as median fluorescence intensity (MFI) minus background (MFI-bg). Background subtraction of raw MFI values was accomplished using blank wells on each assay plate containing Buffer B only. Assay plates contained a negative control, as well as a positive pool serum control. The positive pool serum control was serially diluted to generate an 8-point curve that covered the linear MFI range for most of the antigens on the multiplex panel. Average reactivities of the antigens with these control samples were used to generate plate data acceptance/rejection criteria as a measure of plate-to-plate variation over time. The cutoff for the SAG2A antigen was determined by testing a panel of positive and negative sera (*N* = 16) on MBA at the U.S. CDC that were characterized using the Sabin–Feldman dye binding assay. The average MFI-bg of the highest negative sample value and lowest positive sample value was chosen as the cutoff value. A high titer serum sample was serially diluted 2-fold with 8 points and run at both sites to generate a standard curve with arbitrary units (AU) corresponding to MFI-bg. The standard curve was then used to calculate a cutoff at the Nigeria Center for Disease Control National Reference Laboratory that would correspond to the back-calculated AU of the U.S. CDC cutoff value. This process was done for both SAG2A couplings used in this analysis. For tranches 1 and 2, *T. gondii* seropositivity was defined as MFI-bg >211, and the cutoff for tranche 3 was defined as MFI-bg >344 (because different bead couplings were used). Only samples with a bead count >20 were included in this analysis.

## STATISTICAL ANALYSES

We performed all statistical analyses in R (version 4.2.2; R Core Team 2022)^18^ and created maps in QGIS (version 3.22.6; QGIS Association). Study weights were constructed to account for selection and nonresponse at the different sampling stages and tranches and to preserve substratum population totals for 5-year age groups, sex, and state. This was achieved by adjusting blood draw weights from NAIIS. Because the latter were derived from NAIIS household weights for children and adults, reweighting respected sampling and clustering design. We calculated seroprevalence estimates, along with confidence intervals, with survey design weight using the survey R (v4.1.1; Lumley 2020), and the epikit R package (v0.1.5; Spina and Schumacher 2023).^19^^,^^20^ We also performed descriptive analysis of seroprevalence by sociodemographic groups.

The NAIIS survey data collection enabled the analysis of various risk factors for *T. gondii* seropositivity. We performed bivariate analyses, calculating crude odds ratios (ORs) with 95% CIs, and considered *P* <0.05 as statistically significant. For the multivariate logistic regression model, we used a *P* cutoff of ≤0.20 from the bivariate analyses to select candidate variables. This decision was made to ensure important variables were not overlooked.^21^ The parent variable of whether a household owned livestock, herds, other farm animals, camels, or poultry was chosen for the multivariate model instead of variables assessing individual animal species ownership.

Collinearity was assessed using the generalized variance inflation factor (GVIF) and degrees of freedom (DF). We calculated the adjusted GVIF (GVIF^(1/(2*DF))), with values under 2 considered acceptable.^22^ Our assessment revealed no problematic collinearity among the variables.

We used stepwise selection to build the final multivariable model, selecting the model with the lowest Akaike information criterion value. The following factors were used in the multivariate logistic regression model: age group, pregnancy status, wealth quintile, education level, urban/rural residence, number of household members, whether the household owned any livestock, and improved/unimproved household source of drinking water and toilet facility. Data on household sources of drinking water and toilet facilities were categorized as “improved” or “unimproved” according to definitions from the WHO and UNICEF.^23^ The multivariate analyses produced adjusted ORs (aORs) and 95% CIs, with *P* <0.05 considered statistically significant. Survey weights were incorporated into both the bivariate and multivariate analyses.

## RESULTS

A total of 44,269 WRA had SAG2A laboratory results available. A majority of WRA were between 25 and 44 years old (26,841, 55.5%), were not pregnant at the time of the survey (39,597, 90.7%), lived in households with 1 to 10 members (38,197, 86.3%), had access to improved drinking water sources (32,216, 78.7%) and toilet facilities (24,257, 61.0%), and did not treat drinking water to make it safer (40,533, 90.1%). Many WRA had secondary education (18,021, 45.6%). Approximately equal proportions of WRA reported being Christian or Muslim, having an urban or rural place of residence, and residing in a household that owned or did not own livestock, herds, other farm animals, camels, or poultry (Table 1).

**Table 1:** Bivariate and multivariate analysis of demographic characteristics and risk factors associated with *Toxoplasma gondii* exposure for women of reproductive age (15–44 years of age)—Nigeria, 2018

Variable	Total No. of Participants*	Weighted Proportion (%) of All Participants (95% CI)	No. of Seropositive Participants	Weighted Prevalence of Seropositivity (%) (95% CI)	OR (95% CI)	*P*	aOR (95% CI)	*P*
Total	44,269	–	12,148	26.8 (25.8–27.7)	–	–	–	–
Age in years								
15–24	17,428	44.5 (43.8–45.3)	3,831	21.2 (20.1–22.4)	Ref	–	Ref	–
25–44	26,841	55.5 (54.7–56.2)	8,317	31.2 (30.1–32.3)	**1.68 (1.56–1.82)**	**<0.001**	**1.74 (1.60–1.90)**	**<0.001**
Pregnancy status								
Pregnant	4,078	9.3 (8.6–10.1)	983	23.9 (21.8–26.2)	Ref	–	Ref	–
Not pregnant	39,597	90.7 (89.9–91.4)	10,965	27.0 (26.0–27.9)	**1.17 (1.04–1.32)**	**0.01**	1.11 (0.98–1.27)	0.11
Unknown^†^	594	–	203	–	–	–	–	–
Religion								
Christian	24,888	48.7 (46.4–50.9)	7,631	31.6 (30.5–32.7)	Ref	–	NA^‡^	NA^‡^
Muslim	19,133	51.3 (49.1–53.6)	4,442	22.3 (21.0–23.7)	**0.62 (0.56–0.68)**	**<0.001**	NA^‡^	NA^‡^
No religion/other/traditional^†^	248	–	77	–	–	–	NA^‡^	NA^‡^
Household’s wealth quintile								
Lowest	9,388	18.4 (16.0–21.1)	1,513	15.2 (13.7–16.9)	Ref	–	Ref	–
Second	9,376	18.0 (16.3–22.4)	2,131	20.4 (18.9–21.9)	**1.42 (1.22–1.67)**	**<0.001**	**1.48 (1.24–1.76)**	**<0.001**
Middle	9,380	20.5 (18.9–22.4)	2,873	29.4 (27.5–31.3)	**2.31 (1.95–2.75)**	**<0.001**	**2.59 (2.13–3.15)**	**<0.001**
Fourth	8,702	21.1 (19.3–23.1)	3,062	33.2 (31.4–35.0)	**2.77 (2.37–3.23)**	**<0.001**	**3.33 (2.73–4.06)**	**<0.001**
Highest	7,423	21.9 (20.0–23.9)	2,569	34.0 (32.3–35.7)	**2.87 (2.47–3.33)**	**<0.001**	**3.87 (3.13–4.79)**	**<0.001**
Education								
No education	10,785	24.6 (22.4–26.8)	2,290	19.5 (18.1–21.1)	Ref	–	Ref	–
Primary	7,860	18.2 (16.9–19.5)	2,438	31.2 (29.3–33.1)	**1.87 (1.65–2.12)**	**<0.001**	**1.36 (1.19–1.6)**	**<0.001**
Secondary	18,021	45.6 (43.7–47.5)	5,389	29.7 (28.5–30.9)	**1.74 (1.56–1.95)**	**<0.001**	**1.19 (1.05–1.35)**	**0.01**
Tertiary	4,726	11.7 (10.5–13.0)	1,408	29.5 (27.6–31.6)	**1.73 (1.51–1.99)**	**<0.001**	0.91 (0.78–1.04)	0.26
Unknown^†^	2,877	–	623	–	–	–	–	–
Location of residence								
Rural	27,423	51.6 (47.8–55.4)	7,082	24.8 (23.7–26.1)	Ref	–	Ref	–
Urban	16,846	48.4 (44.6–52.2)	5,066	28.9 (27.5–30.4)	**1.23 (1.10–1.37)**	**<0.001**	**0.85 (0.75–0.95)**	**0.01**
Total household members								
1–10	38,197	86.3 (84.7–87.7)	10,804	27.6 (26.7–28.6)	Ref	–	Ref	–
11–20	5,611	12.9 (11.5–14.4)	1,249	21.7 (19.7–23.8)	**0.73 (0.64–0.82)**	**<0.001**	**0.86 (0.75–0.98)**	**0.04**
>20	461	0.8 (0.5–1.2)	95	20.1 (13.1–29.8)	0.66 (0.38–1.14)	0.12	0.80 (0.44–1.46)	0.50
Animal ownership: household owns livestock, herds, other farm animals, camels, or poultry								
No	20,816	52.1 (49.5–54.6)	6,716	30.7 (29.5–31.9)	Ref	–	Ref	–
Yes	23,396	47.9 (45.4–50.5)	5,408	22.8 (21.6–24.0)	**0.66 (0.61–0.73)**	**<0.001**	**0.83 (0.76–0.91)**	**<0.001**
Unknown^†^	57	–	24	–	–	–	–	–
Drinking water								
Household source of drinking water								
Improved^‡^	32,216	78.7 (76.0–81.1)	9,248	27.8 (26.7–28.9)	Ref	–	Ref	–
Unimproved^§^	11,829	21.3 (18.9–24.0)	2,847	23.1 (21.3– 25.0)	**0.78 (0.69–0.89)**	**<0.001**	**1.19 (1.04–1.37)**	**0.01**
Unknown^†^	224	–	53	–	–	–	–	–
Household treats water to make it safer to drink								
No	40,533	90.1 (88.7–91.3)	11,098	26.7 (25.7–27.6)	Ref	–	NA	NA
Yes	3,619	9.9 (8.7–11.3)	1,025	27.7 (25.2–30.4)	1.05 (0.92–1.21)	0.45	NA	NA
Unknown^†^	117	–	25	–	–	–	NA	NA
Sanitation: type of toilet facility								
Improved^ǁ^	24,257	61.0 (58.1–63.9)	6,996	27.5 (26.4–28.7)	Ref	–	Ref	–
Unimproved^¶^	19,519	39.0 (36.1–41.9)	4,933	25.4 (24.0–26.7)	**0.89 (0.82–0.98)**	**0.02**	**1.36 (1.23–1.61)**	**<0.001**
Unknown^†^	493	–	219	–	–	–	–	–

aOR = adjusted odds ratio; NA = not applicable; OR = odds ratio.

* Total number of participants for whom SAG2A data is available.

^†^ Excluded from calculation of weight proportion of all participants and weighted prevalence of seropositivity.

^‡^ Improved household source of drinking water: piped into dwelling, piped to yard/plot, public tap/standpipe, piped to neighbor, tube well or borehole, protected well, protected spring, rainwater, tanker truck, cart with small tank/jerry can/cartless vendor, bottled water/dispenser water, sachet (pure) water.

^§^ Unimproved household source of drinking water: unprotected well, unprotected spring, surface water (river/dam/lake/pond/stream/canal/irrigation channel).

^ǁ^ Improved toilet facility: flush to piped sewer system, flush to septic tank, flush to pit latrine, flush (do not know where), ventilated improved pit latrine, pit latrine with slab, composting toilet.

^¶^ Unimproved toilet facility: flush to somewhere else, bucket toilet, hanging toilet, no facility, pit latrine.

Bold text indicates *P* < 0.05.

**Figure 1.**
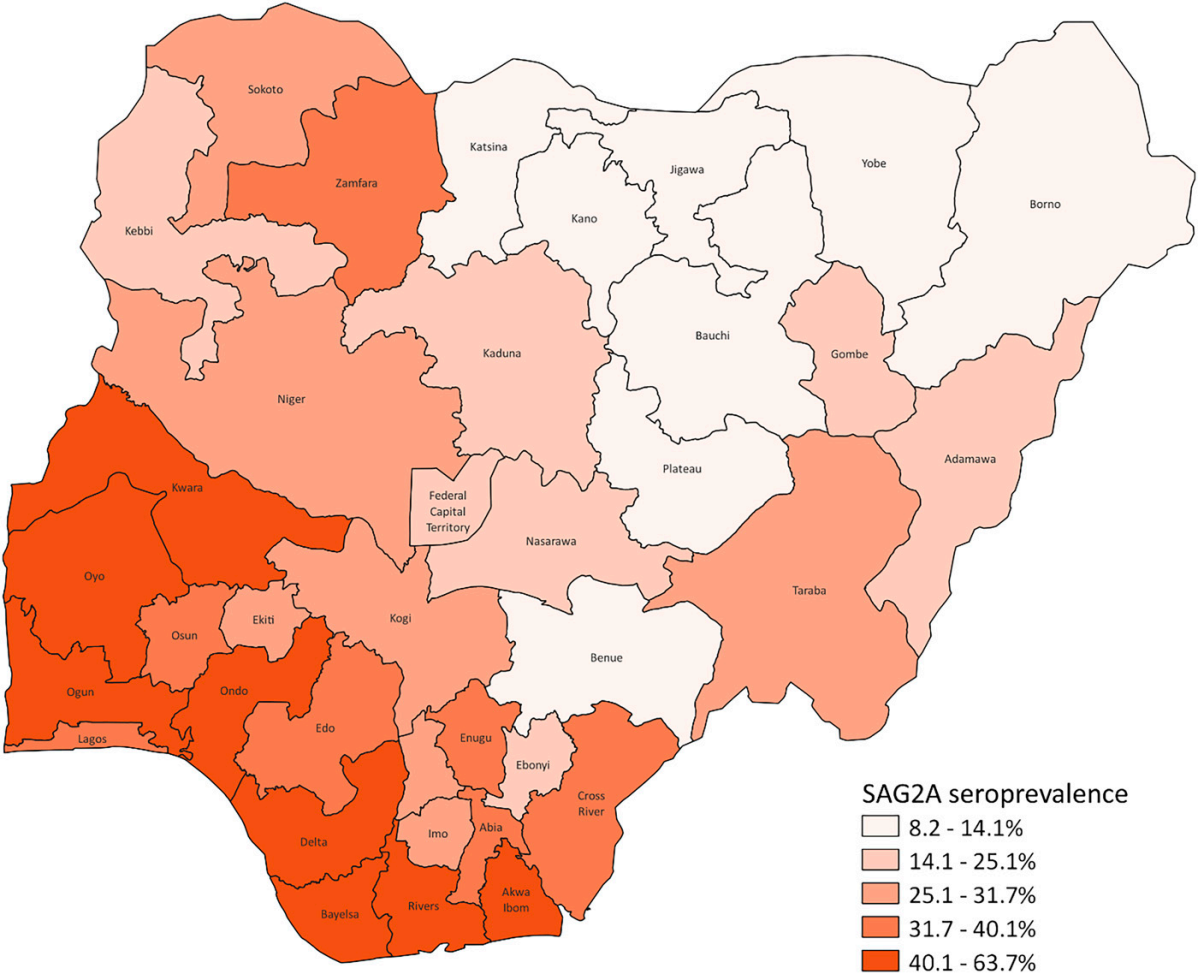
SAG2A seroprevalence by state in women of reproductive age (15–44 years)—Nigeria, 2018.

Anti-SAG2A seropositivity overall was 26.8% (95% CI: 25.8–27.7%) (Table 1). Evidence of *T. gondii* exposure was found in WRA from all 36 states and the FCT; seroprevalence ranged from 8.2% (95% CI: 5.5–12.0%) in Yobe in the North East Zone to 63.7% (95% CI: 58.5–68.5%) in Bayelsa in the South South Zone (Table 2; Figure 1). Seroprevalence was generally higher in the southern part of Nigeria (South East Zone: 30.6%, 95% CI: 28.7–32.6%; South South Zone: 44.0%, 95% CI: 42.3–45.7%; South West Zone: 39.1%, 95% CI: 37.1–41.2%) compared with the northern part of Nigeria (North Central Zone: 24.4%, 95% CI: 22.2–26.7%; North East Zone: 14.4%, 95% CI: 12.9–16.0%; North West Zone: 16.7%, 95% CI: 15.0–18.6%) (Table 2).

**Table 2:** Weighted prevalence of IgG seropositivity to *Toxoplasma gondii* antigen SAG2A by geopolitical zone and state—Nigeria, 2018

Geopolitical Zone and State	Total No. of Participants with Available SAG2A Data	Weighted Proportion (%) of All Participants (95% CI)	No. of Seropositive Participants	Weighted Prevalence of Seropositivity (%) (95% CI)
North-Central Zone	8,834	13.4 (12.2–14.6)	1,772	24.4 (22.2–26.7)
Benue	1,908	2.5 (2.3–2.8)	193	10.3 (7.9–13.3)
FCT	903	1.0 (0.7–1.3)	231	25.0 (22.3–27.9)
Kogi	337	2.0 (1.5–2.7)	103	30.1 (23.6–37.4)
Kwara	593	1.1 (0.9–1.4)	301	51.4 (45.1–57.7)
Nasarawa	2,308	1.2 (1.1–1.4)	524	22.7 (20.3–25.4)
Niger	457	3.4 (2.7–4.5)	144	31.3 (25.3–38.0)
Plateau	2,328	2.1 (1.9–2.3)	276	10.9 (9.0–13.2)
North-East Zone	7,414	14.2 (13.1–15.4)	1,089	14.4 (12.9–16.0)
Adamawa	877	2.2 (1.7–2.7)	181	19.7 (16.7–23.0)
Bauchi	2,661	4.2 (3.8–4.6)	249	9.5 (8.0–11.3)
Borno	534	3.2 (2.5–4.1)	72	13.8 (9.7–19.3)
Gombe	2,199	1.5 (1.3–1.7)	350	15.5 (13.3–18.0)
Taraba	734	1.6 (1.4–2.0)	195	26.0 (21.4–31.2)
Yobe	409	1.5 (1.2–1.9)	42	8.2 (5.5–12.0)
North-West Zone	8,876	29.0 (26.7–31.4)	1,836	16.7 (15.0–18.6)
Jigawa	2,179	3.2 (2.9–3.6)	217	9.5 (8.4–10.7)
Kaduna	1,104	5.2 (4.2–6.4)	202	18.5 (14.1–23.9)
Kano	469	9.3 (7.2–11.7)	61	13.0 (9.4–17.6)
Katsina	286	4.7 (3.4–6.4)	31	11.3 (7.8–15.9)
Kebbi	1,931	2.1 (1.9–2.3)	451	22.5 (19.2–26.1)
Sokoto	1,771	2.5 (2.2–2.8)	467	25.2 (22.2–28.5)
Zamfara	1,136	2.1 (1.9–2.3)	407	36.3 (31.6–41.2)
South-East Zone	6,745	11.6 (10.6–12.6)	1,857	30.6 (28.7–32.6)
Abia	531	2.2 (1.7–2.7)	189	34.0 (27.5–41.2)
Anambra	1,585	2.6 (2.3–3.0)	501	31.0 (28.6–33.4)
Ebonyi	2,912	1.5 (1.4–1.7)	620	20.7 (18.7–22.9)
Enugu	539	2.8 (2.2–3.5)	190	35.5 (31.3– 39.9)
Imo	1,178	2.5 (2.1–2.9)	357	27.9 (24.6–31.4)
South-South Zone	8,328	12.3 (11.6–13.1)	3,894	44.0 (42.3–45.7)
Akwa Ibom	1,822	2.4 (2.2–2.6)	1,006	54.6 (51.7–57.4)
Bayelsa	836	0.8 (0.7–1.0)	538	63.7 (58.5–68.5)
Cross River	1,949	2.0 (1.8–2.3)	766	36.5 (32.8–40.2)
Delta	1,605	2.0 (1.9–2.2)	767	46.6 (42.7–50.5)
Edo	776	1.7 (1.4–2.0)	257	32.0 (26.5–37.9)
Rivers	1,340	3.4 (3.0–3.9)	560	40.8 (37.4–44.3)
South-West Zone	4,072	19.5 (18.2–21.0)	1,700	39.1 (37.1–41.2)
Ekiti	210	1.0 (0.8–1.4)	60	25.9 (19.2–34.0)
Lagos	902	8.4 (7.5–9.5)	360	37.4 (33.8–41.0)
Ogun	372	2.7 (2.1–3.5)	164	43.3 (38.7–48.0)
Ondo	578	2.0 (1.6–2.5)	250	44.3 (38.5–50.3)
Osun	306	1.9 (1.5–2.5)	110	35.7 (29.5–42.4)
Oyo	1,704	3.4 (3.1–3.8)	756	42.9 (40.0–45.9)

FCT = Federal Capital Territory.

Seropositivity was higher in 25- to 44-year-olds (31.2%, 95% CI: 30.1–32.3%) than in 15–24-year-olds (21.2%, 95% CI: 20.1–22.4%), and the bivariate analysis demonstrated that there were greater crude odds of seropositivity associated with being in the 25- to 44-year age group (OR: 1.68, 95% CI: 1.56–1.82) than in the 15- t0 24-year age group. Seropositivity was 23.9% in pregnant (95% CI: 21.8–26.2%) and 27.0% in nonpregnant women (95% CI: 26.0–27.9%); compared with pregnant women, nonpregnant women had a slightly greater crude odds of seropositivity (OR: 1.17, 95% CI: 1.04–1.32). Compared with the lowest wealth quintile, successively greater crude odds of seropositivity were associated with each higher wealth quintile (2nd quintile: OR: 1.42, 95% CI: 1.22–1.67; 3rd quintile: OR: 2.31, 95% CI: 1.95–2.75; 4th quintile: OR: 2.77, 95% CI: 2.37–3.23; highest quintile: OR: 2.87, 95% CI: 2.47–3.33). Compared with no education, there were greater crude odds of seropositivity associated with having primary (OR: 1.87, 95% CI: 1.65–2.12), secondary (OR: 1.74, 95% CI: 1.56–1.95), or tertiary (OR: 1.73, 95% CI: 1.51–1.99) education. The crude odds of seropositivity was also greater among those residing in an urban area (OR: 1.23, 95% CI: 1.10–1.37) compared with a rural area (Table 1).

Decreased crude odds of seropositivity were associated with an unimproved household source of drinking water (OR: 0.78, 95% CI: 0.69–0.89) and toilet facility (OR: 0.89, 95% CI: 0.82–0.98) compared with an improved household source of drinking water and toilet facility; having 11–20 household members (OR: 0.73, 95% CI: 0.64–0.82) compared with 1–10 household members; being Muslim (OR: 0.62, 95% 0.56–0.68) compared with being Christian; and being in a household that owned livestock, herds, other farm animals, camels, or poultry (OR: 0.66, 95% CI: 0.61–0.73) compared with a household with no animal ownership (Table 1). We also examined the specific types of animals owned and, where statistically significant, found that they were all associated with decreased crude odds of seropositivity (data not shown). Goats were more commonly owned by households (compared with other animals such as cattle or sheep), with the vast majority of households that owned goats owning 10 or fewer goats. As such, examining the relationship between the specific number of goats owned by the household and seropositivity was not expected to be helpful.

There was no statistical difference in crude odds of seropositivity by whether the household treated water to make it safer to drink (*P* = 0.45) (Table 1).

Multivariate analyses revealed that greater adjusted odds of seropositivity were associated with being in the 25- to 44-year age group (aOR: 1.74, 95% CI: 1.60–1.90) compared with the 15- to 24-year age group; being in higher wealth compared with the lowest wealth quintile (2nd quintile: aOR: 1.48 95% CI: 1.24–1.76; 3rd quintile: aOR: 2.59, 95% CI: 2.13–3.15; 4th quintile: aOR: 3.33, 95% CI: 2.73–4.06; highest quintile: aOR: 3.87, 95% CI: 3.13–4.79); having a primary (aOR: 1.36, 95% CI: 1.19–1.64) or secondary (aOR: 1.19, 95% CI: 1.05–1.35) education compared with no education, and having an unimproved household source of drinking water (aOR: 1.19, 95% CI: 1.04–1.37) or toilet facility (aOR: 1.36, 95% CI: 1.23–1.61) compared with an improved household source of drinking water and toilet facility (Table 1).

Lower adjusted odds of seropositivity were associated with residing in an urban area (aOR: 0.85, 95% CI: 0.75–0.95) compared with a rural area, having 11–20 household members (aOR: 0.86, 95% CI: 0.75–0.98) compared with 1–10 household members, and being in a household that owned livestock, herds, other farm animals, camels, or poultry (aOR: 0.83, 95% CI: 0.76–0.91) compared with a household with no animal ownership (Table 1).

There was no statistical difference in adjusted odds of seropositivity by pregnancy status (*P* = 0.11) (Table 1).

## DISCUSSION

An overall anti-SAG2A IgG seropositivity in WRA of 26.8% (95% CI: 25.8–27.7%) in this sample is lower than the pooled prevalence estimate of *T. gondii* infection among pregnant women of 40.25% provided by a prior systematic review which examined studies across 17 Nigerian states.^7^ This is Nigeria’s first population-based estimate of *T. gondii* exposure in WRA, obtained by leveraging MBA to test specimens from a prior survey. To the authors’ knowledge, no other nationally representative population-based estimates for *T. gondii* seroprevalence exist for other countries in sub-Saharan Africa. The NAIIS is a population-based survey, and our study included all WRA (not just pregnant women) who consented to specimen storage and future testing, providing us with a high level of confidence in the accuracy of our seroprevalence estimate, as evidenced by its narrow confidence interval. Women who have been infected with *T. gondii* (i.e., IgG seropositive) before pregnancy have minimal risk of transmitting the infection to their fetus in subsequent pregnancies. As such, this study indicates that almost 75% of WRA are at risk of congenital transmission of *T. gondii* if infected during a future pregnancy.

Our results also provide some preliminary insights into risk factors for *T. gondii* exposure among WRA in Nigeria. As expected, seroprevalence was higher in the 25- to 44-year age group than in 15- to 24-year-olds because older WRA have had more opportunities for exposure and IgG antibodies against *T. gondii* usually persist for life.^24^ Generally, the seroprevalence of toxoplasmosis in WRA was higher in the southern part of Nigeria than in the northern part. This corroborates a similar finding from a previous study that examined *T. gondii* prevalence across 17 states in Nigeria, which demonstrated that there was geographic variation in *T. gondii* infection, with the highest prevalence observed in the south-south region.^7^ This observation may reflect the different climate zones found in Nigeria: a tropical monsoon climate in the south, a tropical savannah climate in most of the central region, and a hot and semiarid climate in the north. The prevalence of *T. gondii* worldwide is higher in humid tropical areas and lower in hot and dry areas such as northern Nigeria, where oocysts may be less likely to remain viable, thus decreasing their infectivity.^25^ In the south, annual rainfall amounts are usually more than 2,000 mm, whereas there is typically only 500–700 mm of rain in the north.^26^ In 2021, the annual mean maximum temperature was 34.0–37.0°C in the north compared with 28.0 to 30.0°C in the south.^27^ Almost 50% of toxoplasmosis cases globally are estimated to result from foodborne transmission, and toxoplasmosis is one of the most common foodborne parasitic diseases worldwide.^28^ The higher seroprevalence of toxoplasmosis in WRA in the southern part of Nigeria may also be related to farming that predominantly occurs in southern Nigeria compared with herding in northern Nigeria, as farming activities such as tilling soil and harvesting crop may be associated with accidental ingestion of soil that may contain infective oocysts.^29^ Another contributing factor may be regional differences in meat consumption. Meat such as pork, beef, lamb, and chicken are reportedly eaten by more Nigerian households in southern regions than in northern regions.^30^ Likewise, more Nigerian households in more affluent wealth groups are reported to eat more meat such as pork, beef, lamb, and chicken than those in poorer wealth groups,^30^ which may explain why those in higher wealth quintiles had greater odds of seropositivity compared with the lowest. The NAIIS questionnaires did not collect information on meat consumption or cooking habits, although meat is generally well cooked in Nigeria. However, greater meat consumption means more opportunities to eat undercooked meat that may harbor tissue cysts, resulting in foodborne transmission of *T. gondii*.

Unimproved drinking water sources were also associated with higher odds of seropositivity. Although this study did not examine specific reasons for this, unimproved water sources, which include unprotected wells, unprotected springs, and surface water, may become contaminated with oocysts shed by felids. This has been observed in other countries, where recorded outbreaks of toxoplasmosis have been associated with ingesting water contaminated with oocysts. For example, in 1995, an outbreak of acute toxoplasmosis in Canada was found to be associated with a municipal water system supplied by a reservoir that was likely contaminated with *T. gondii* oocysts through increased runoff in the watershed where domestic and feral cats and cougars were known to be present. Several felids trapped in the area were found to be seropositive for *T. gondii*.^31^ Similarly, an outbreak of ocular toxoplasmosis was identified in 2004 in India, where most cases were in an area supplied by one water reservoir that, before the outbreak, may have been contaminated due to heavy rainfall in catchment areas known to be inhabited by domestic and wild cats.^32^ Multiple outbreaks of toxoplasmosis in Brazil have also been associated with drinking water contaminated with *T. gondii* oocysts.^33^^–^^35^

Some of our findings are less easily explained. Although this study indicates that unimproved toilet facilities are associated with higher odds of seropositivity, humans are not exposed to *T. gondii* through human urine or feces. Prior studies in Mexico have demonstrated increased *T. gondii* seropositivity associated with the use of latrines (as opposed to toilet facilities with sewage pipes).^36^^,^^37^ Improved toilet facilities may instead be a surrogate for another unmeasured factor such as handwashing, which has been previously demonstrated to be protective against toxoplasmosis, particularly after contact with contaminated soil or before eating.^38^^–^^41^ In Ethiopia, households with improved sanitation facilities were twice as likely to have basic handwashing facilities than those with unimproved sanitation facilities.^42^ As such, it is possible that improved toilet facilities in Nigeria are also more likely to have handwashing facilities. However, the toilet facility variable collected in NAIIS does not specifically examine handwashing facilities or practices, and handwashing was also not queried in the NAIIS, so we could not test this hypothesis. Additionally, there were lower odds of seropositivity to *T. gondii* for those residing in an urban area compared with a rural area. This corroborates previous studies from other countries that have demonstrated an association between increased toxoplasmosis seroprevalence rates and living in rural areas.^38^^,^^43^^,^^44^ Other studies also indicate that rural residence may be associated with poorer access to healthcare, a lower level of health literacy, unimproved sanitation facilities, and increased soil or animal contact, resulting in greater exposure to the environment, which may contribute to transmission of *T. gondii*.^43^^,^^44^ These rural environments may also be more likely to be contaminated with *T. gondii* oocysts.^43^^,^^44^

It is also difficult to explain why our study demonstrates that having primary and secondary education would result in greater odds of seropositivity to *T. gondii* compared with having no formal education because prior studies suggest that increased odds of *T. gondii* seropositivity is associated with a lower education level^36^^,^^37^^,^^45^^–^^49^ or that there is no significant association with education level.^50^^–^^54^ It could be that people with more education are also in higher wealth quintiles, who are also more likely to consume meat (and had greater odds of seropositivity), but no collinearity was demonstrated between factors used in the multivariate logistic regression model including education and wealth quintile. Our study revealed that individuals living in a household with 11–20 members were less likely to be seropositive for *T. gondii* than those in a household with 1–10 members. Interpretation of this observation is challenging. Only a few prior studies from other countries have examined crowding in relation to toxoplasmosis seropositivity.^37^^,^^46^ However, the number of household members may not correlate with crowding in this setting. Women of reproductive age in households that owned livestock, herds, other farm animals, camels, or poultry appear to have lower odds of seropositivity for *T. gondii* than those in households that do not. It is difficult to explain this observation, particularly because prior studies indicate that occupational exposure to livestock (e.g., farmers and abattoir workers) is associated with a higher seroprevalence of *T. gondii*.^52^^,^^55^ It is possible that other factors not examined in this study such as whether livestock ownership in Nigeria is correlated with less exposure to soil (e.g., not performing farming activities such as tilling) could have contributed to these findings.

Because the NAIIS was designed specifically for HIV and not toxoplasmosis, data on specific risk factors for toxoplasmosis (e.g., consumption of undercooked meat, cat ownership, or potential for contamination of drinking water sources with cat feces) were not collected, limiting interpretation. There may also be unmeasured confounders for which the analyses performed could not account. Previous studies on targeted populations in Nigeria demonstrate mixed results on whether there is a significant association between *T. gondii* seropositivity and contact with cats.^47^^,^^50^^,^^52^ The NAIIS was designed and implemented to make the survey as nationally representative as possible, but assumptions based on the use of outdated population statistics during sampling, or any deviations from protocol implementation, may limit generalizability of the findings to the Nigerian population. For example, 72 (1.8%) EAs out of a total of 4,035 in the NAIIS could not be visited because of security challenges, thus potentially limiting the representativeness of survey estimates in these areas. One EA was not visited due to flooding. In addition, although the balance of sampling and household clustering effects was closely adhered to, blood draw weights in NAIIS were ultimately derived from household weights, and thus some caution is necessary when interpreting results.

This study highlights the benefits of using specimens collected for previous surveys to investigate other diseases of public health importance. This understanding of the national prevalence of *T. gondii* infection in WRA in Nigeria may help determine public health priorities and also allow for the monitoring of changes over time, particularly if prevention efforts are implemented. These results provide some preliminary insights for developing more targeted disease control and prevention measures—for example, health education programs during routine antenatal care of pregnant women and increasing awareness in healthcare providers. Such efforts may help reduce the risk of infection during pregnancy, congenital transmission of *T. gondii,* and adverse health outcomes in infants.

## CONCLUSION

Almost 75% of WRA in Nigeria in 2018 were at risk for congenital transmission of *T. gondii* if infected during a future pregnancy. We found that WRA from all states had *T. gondii* exposure. Seroprevalence was higher in 25- to 44-year-olds than in 15- to 24-year-olds. A similar proportion of pregnant and nonpregnant women were seropositive. Decreased odds of seropositivity were associated with living in an urban area and owning livestock. Increased odds of seropositivity were associated with unimproved toilet facilities and drinking water sources, being in a higher wealth quintile, and primary and secondary education compared with no education.

Prevention of primary toxoplasmosis infection in pregnant women will help prevent congenital transmission. Women of reproductive age should be educated on measures that they can take particularly during pregnancy, to reduce foodborne and environmental transmission of toxoplasmosis, such as not eating undercooked meat, reducing exposure to oocysts from the environment, and handwashing. Future studies could also further investigate potential exposure risk factors to inform the development of effective prevention measures. Future studies could also examine the possible reasons for a higher prevalence of *T. gondii* in southern Nigeria. Although the data do not include information on congenital transmission of *T. gondii*, future studies could evaluate the prevalence of congenital toxoplasmosis in the country.
